# Assessing structural variation in a personal genome—towards a human reference diploid genome

**DOI:** 10.1186/s12864-015-1479-3

**Published:** 2015-04-11

**Authors:** Adam C English, William J Salerno, Oliver A Hampton, Claudia Gonzaga-Jauregui, Shruthi Ambreth, Deborah I Ritter, Christine R Beck, Caleb F Davis, Mahmoud Dahdouli, Singer Ma, Andrew Carroll, Narayanan Veeraraghavan, Jeremy Bruestle, Becky Drees, Alex Hastie, Ernest T Lam, Simon White, Pamela Mishra, Min Wang, Yi Han, Feng Zhang, Pawel Stankiewicz, David A Wheeler, Jeffrey G Reid, Donna M Muzny, Jeffrey Rogers, Aniko Sabo, Kim C Worley, James R Lupski, Eric Boerwinkle, Richard A Gibbs

**Affiliations:** Human Genome Sequencing Center, Baylor College of Medicine, Houston, TX 77030 USA; Department of Molecular and Human Genetics, Baylor College of Medicine, Houston, TX 77030 USA; DNAnexus, Mountain View, CA 94040 USA; Spiral Genetics Inc, Seattle, WA 98117 USA; BioNano Genomics Inc, San Diego, CA 92121 USA; Collaborative Innovation Center of Genetics and Development, School of Life Sciences, Fudan University, Shanghai, 200438 China; Department of Pediatrics, Baylor College of Medicine, Houston, TX 77030 USA; Texas Children’s Hospital, Houston, TX 77030 USA; Human Genetics Center, University of Texas Health Science Center at Houston, Houston, TX 77030 USA

**Keywords:** Structural variation, Long-read sequencing, SV software

## Abstract

**Background:**

Characterizing large genomic variants is essential to expanding the research and clinical applications of genome sequencing. While multiple data types and methods are available to detect these structural variants (SVs), they remain less characterized than smaller variants because of SV diversity, complexity, and size. These challenges are exacerbated by the experimental and computational demands of SV analysis. Here, we characterize the SV content of a personal genome with Parliament, a publicly available consensus SV-calling infrastructure that merges multiple data types and SV detection methods.

**Results:**

We demonstrate Parliament’s efficacy via integrated analyses of data from whole-genome array comparative genomic hybridization, short-read next-generation sequencing, long-read (Pacific BioSciences RSII), long-insert (Illumina Nextera), and whole-genome architecture (BioNano Irys) data from the personal genome of a single subject (HS1011). From this genome, Parliament identified 31,007 genomic loci between 100 bp and 1 Mbp that are inconsistent with the hg19 reference assembly. Of these loci, 9,777 are supported as putative SVs by hybrid local assembly, long-read PacBio data, or multi-source heuristics. These SVs span 59 Mbp of the reference genome (1.8%) and include 3,801 events identified only with long-read data. The HS1011 data and complete Parliament infrastructure, including a BAM-to-SV workflow, are available on the cloud-based service DNAnexus.

**Conclusions:**

HS1011 SV analysis reveals the limits and advantages of multiple sequencing technologies, specifically the impact of long-read SV discovery. With the full Parliament infrastructure, the HS1011 data constitute a public resource for novel SV discovery, software calibration, and personal genome structural variation analysis.

**Electronic supplementary material:**

The online version of this article (doi:10.1186/s12864-015-1479-3) contains supplementary material, which is available to authorized users.

## Background

Analysis of personal genome sequence variation data is currently dominated by single-nucleotide variant (SNV) and small insertion and deletion (indel) detection. Such variants are easily visualized, relatively straightforward to detect, and have driven many successful studies of the associations between genomic variation and human disease [1-3]. Larger variants, however, account for a greater number of variable bases in the genome, with up to 13% of the human genome subject to such variation [4]. These structural variants (SVs) include copy-number variants (CNVs), copy-number neutral inversions, mobile element insertions (MEIs), deletions, translocations, and complex combinations of these events. Given their scope, it is unsurprising that SVs have been implicated in a broad variety of diseases and are thought to contribute greatly to human genetic and phenotypic diversity [5]. Nevertheless, structural variation remains less understood and more challenging to detect and characterize than SNVs and smaller indels, both across populations and in personal genomes. As whole-genome sequencing (WGS) becomes increasingly employed as a research and molecular diagnostic tool, complete and accurate characterization of human genomic variation, including SVs, will be essential to informing clinical decision making.

CNVs, as represented by deviations from the normal diploid state, are the most thoroughly studied class of SVs, with extensive evidence for their role in human health and disease [6-8]. When encompassing genic regions, CNVs can alter the dosage and regulation of their constituent genes, while non-genic CNVs can affect the expression of proximal genes [4,9,10]. Moreover, genic CNVs can contribute to recessive carrier states [11,12] or bring about disease in combination with SNVs on alleles in *trans* [3,13-17]. However, the resolution of CNV loci derived from array-based data is limited by probe density. Read-depth analysis of whole-exome sequence (WES) data has proven comparable to array-based CNV detection methods, but WES CNV calls still lack base-pair resolution of breakpoint junctions [18]. High-resolution SV breakpoint determination is necessary to understanding the disruptive (as opposed to dosage) effects of SVs when their breakpoints fall within functional genomic elements [19], to identifying “mutational signatures” of SV formation mechanisms [20], and to obtain both orientation and genomic positional information for CNV gains.

The availability of NGS data has resulted in a menagerie of SV-detection tools reflecting the broad size range, diversity, and complexity of SVs [21]. These SV-detection methods are often limited by algorithm design, by the underlying data, and restricted to analysis of SVs of a certain type, location, or size. Recent efforts to address these limitations integrate multiple methods (e.g., paired-end, split-read, read-depth, and reference-sequence techniques) to identify consensus SVs [8,22-24]. While such consensus SV callers possess the ability to accommodate various data types and input formats, they are largely designed to call SVs from the most ubiquitous type of sequence data, paired-end (PE) reads, which are generally shorter (~100 bp) than most SVs.

The challenges of SV detection are exacerbated by the lack of a “gold standard” description of structural variation within a personal genome—a reference diploid genome does not exist. Here we combine PE and aCGH data with long-read, long-insert, and whole-genome architecture data from a single individual (HS1011) to improve the scope, resolution, and reliability of SV identification in a personal genome. These data are analyzed via established and newly developed SV discovery tools and then merged and evaluated within Parliament, a SV detection infrastructure designed for multiple data sources and discovery methods. The constituent HS1011 data, the resulting set of SV calls, and the Parliament infrastructure are publicly available for local download and on the cloud-based service DNAnexus, allowing users to compare novel methods to this analysis of HS1011 and readily analyze other data without extensive local compute resources or software expertise.

## Results

### HS1011 SVs

To provide a robust characterization of structural variation in a human personal genome, we examined multiple data sources from a single individual (HS1011). This individual has been previously analyzed with aCGH data and by whole-genome and whole-exome sequencing, revealing novel *SH3TC2* SNVs causative for the subject’s autosomal recessive Charcot-Marie-Tooth (CMT) neuropathy [25,26].

**Figure 1:**
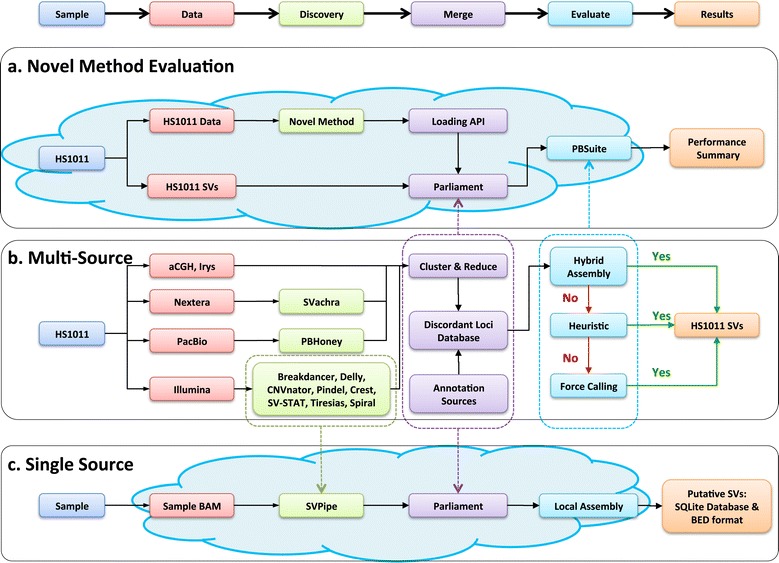
Parliament workflows. The Parliament infrastructure is designed to incorporate multiple data types and software for each data type. **(a)** Novel Method evaluation incorporates new data or methods to the HS1011 workflow. **(b)** The HS1011 workflow. **(c)** The Illumina Only workflow, requiring only a paired-end WGS BAM file as input.

PE sequence and aCGH data were combined with long-read, long-insert size, and genome architecture data to describe the structural variation in the HS1011 genome. Table 1 summarizes the previously collected whole-genome data for HS1011 and the new data specific to this study: a 4.2 million probe aCGH assay, 10X Pacific Biosciences (PacBio) long-read coverage, an Illumina Nextera long-insert library (2X read coverage), and 51X coverage by BioNano Irys single-molecule data. In aggregate, these data represent ~300 billion sequenced nucleotides (~90X) and 7.3 million aCGH probes covering the HS1011 genome. These technologies and their corresponding SV information were next integrated using Parliament, a novel analysis infrastructure (Figure 1b). The SV-detection methods employed by Parliament identify regions of a subject’s genome that are inconsistent with a reference haploid genome assembly. These inconsistencies either can arise from true variation between the subject and reference or else are artifacts of ambiguous mapping between the subject’s reads and reference data.

**Table 1:** **HS1011 data sources**

**Data**	**Type**	**Resolution**	**Source**
WGS Illumina HiSeq	NGS	48X 100x100 bp paired-end	[26]
WGS Illumina Nextera	NGS	2X 100x100 bp 6.5 kbp mate-pair inserts	Methods
WGS SOLiD	NGS	3X 35 bp fragment 10X 25x25 bp paired-end 17X 50x50 bp paired-end	[25,26]
WGS PacBio	Long-Read	10X ~10,000 bp	Methods
Agilent 1 M	aCGH	1-million-probe oligo array	[26]
NimbleGen 2.1 M	aCGH	2.1-million-probe oligo array	[26]
NimbleGen 4.2 M	aCGH	4.2-million-probe oligo array	Methods
Custom Agilent Exon Array	aCGH	44,000 neuropathy-specific oligo array	[26]
BioNano Irys	Genome Mapping	Single-molecule genome architecture	Methods
Sanger-Validated Deletions	Manual	42 fully resolved deletions	Methods

Previously published HS1011 data are indicated with literature references, and data new to the present work are described in Methods.

The Parliament discovery step identified 47,706 initial events in the size range of 100 bp to 1 Mbp that reduced to 31,007 reference-inconsistent loci, spanning 4.8% of the HS1011 genome. To distinguish between structural variants and mapping artifacts, we performed local hybrid assembly with the short- and long-read data (48X Illumina HiSeq and 10X PacBio RS, respectively). Local hybrid assembly supported 7,708 loci, that is, at least one assembled contig supported the predicted SV at a given locus. Recognizing the limitations of local assembly, we then identified 1,103 unassembled loci with support from different technologies and 966 unassembled loci with support from only one technology (excluding PacBio) that are spanned by at least one PacBio read consistent with the predicted SV. In total, these 9,777 SVs had an aggregate length of approximately 93 Mbp that span 59 Mbp of the reference genome (1.8%). Table 2 summarizes the performance of each data source and discovery method that contributes to the HS1011 SVs. The utility of the multi-source approach is immediately apparent, with single data types contributing 68.1% (6,654/9,777) of the SVs. PBHoney, developed specifically to identify SVs from long-read PacBio data [27], alone discovered 3,801 SVs with assembly support, indicating the importance of read length when characterizing structural variation. Additional file 1: Table S1 provides a complete summary of all 31,007 reference-inconsistent loci, which include the 9,777 Parliament SVs and 21,230 unsupported loci.

**Table 2:** **Parliament HS1011 summary**

**Source**	**Method**	**Data**	**Reference**	**Total calls**	**Total Loci**	**Assembled Loci**	**Multi source Loci**	**Force Called Loci**	**Solo assembled**	**Solo forced**
BreakDancer	Paired End	Illumina HiSeq	[43]	6,474	5,520	1,740	335	194	104	82
CNVnator	Read Depth	Illumina HiSeq	[44]	6,232	6,197	679	402	130	176	109
Crest	Split Read	Illumina HiSeq	[45]	2,490	2,219	1,636	138	115	8	3
Delly	Paired End & Split Read	Illumina HiSeq	[23]	4,465	3,720	1,150	323	196	109	97
Pindel	Paired End	Illumina HiSeq	[46]	5,728	4,451	2,432	244	359	421	206
SV-STAT	Reference-guided Assembly	Illumina HiSeq	Methods	893	892	754	90	32	9	1
Tiresias	Consensus Sequences	Illumina HiSeq	Methods	1,354	1,347	269	36	112	76	110
Spiral	Local Assembly	Illumina HiSeq	Methods	1,886	1,881	1,626	100	98	76	14
**Illumina HiSeq Total**				**29,522**	**17,765**	**3,751**	**788**	**814**	**979**	**622**
PBHoney	Local Error and Tail Mapping	PacBio RS	[27]	10,759	10,340	5,883	483	0	3,792	0
SVachra	Discordant Read Pairs	Illumina Nextera	Methods	6,208	4,785	490	454	211	96	211
aCGH + SOLiD	Probe Intensity/Read Depth	aCGH	[25,26]	1,971	1,960	231	452	8	30	8
BioNano Irys	Single-molecule Motif Mapping	Irys	Methods	0	343	201	142	0	41	0
**Total**				**48,460**	**31,184**	**7,733**	**1,133**	**1,033**	**4,897**	**841**

Descriptions and results for each SV-detection method are provided. BioNano Irys data was used only for corroboration, not initial discovery, owing to its large size and propensity to span multiple events.

**Figure 2:**
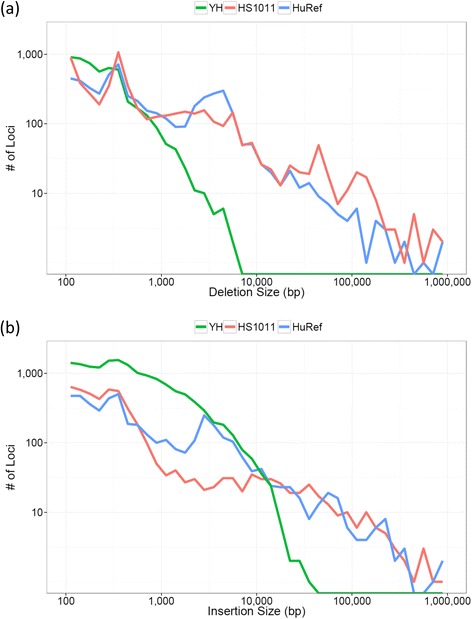
Size distribution. All HS1011 SV events larger than 100 bp and less than 100,000 bp were compared to events from the Venter genome (HuRef) and an Asian Male (YH), both specifically characterized for SV content. In this size regime, the HS1011, HuRef, and YH samples contain 5044, 5127, and 5374 deletions (panel **a**) and 4482, 4479, and 15525 insertions (panel **b**), respectively. The YH SV distributions are based on *de novo* assembly of 35 bp single-end and paired end data. This assembly was used to identify SVs between 1 bp and 50 kbp. Initial events larger than 50 bp were filtered using discordant paired-end mapping of ~35 bp reads. Given the relative abundance of HS1011 sequence data (including both long reads and longer short reads as compared to the YH short reads), and given the differences in methods, it is unlikely that the ~3-fold difference in insertions between the YH set and the HS1011 and HuRef sets represents a significant lack of Parliament sensitivity.

The HS1011 SVs comprised 5,044 deletions, 4,463 insertions, and 270 inversion events. Figure 2 compares the size distributions of HS1011 deletions and insertions with those reported in other personal genomes [28,29]. The HS1011 distributions exhibit peaks at ~300 bp, a characteristic of *Alu* transposon dimorphisms [30]. We assessed all HS1011 events larger than 100 kbp by manually examining the Irys architecture data in the corresponding regions. The Irys data were consistent with 15 events ranging from 100,000 bp to 154,971 bp. Given the resolution and nature of the Irys data, it is unlikely that a large insertion or deletion would not manifest itself in the genomic architecture.

### SV corroboration

**Figure 3:**
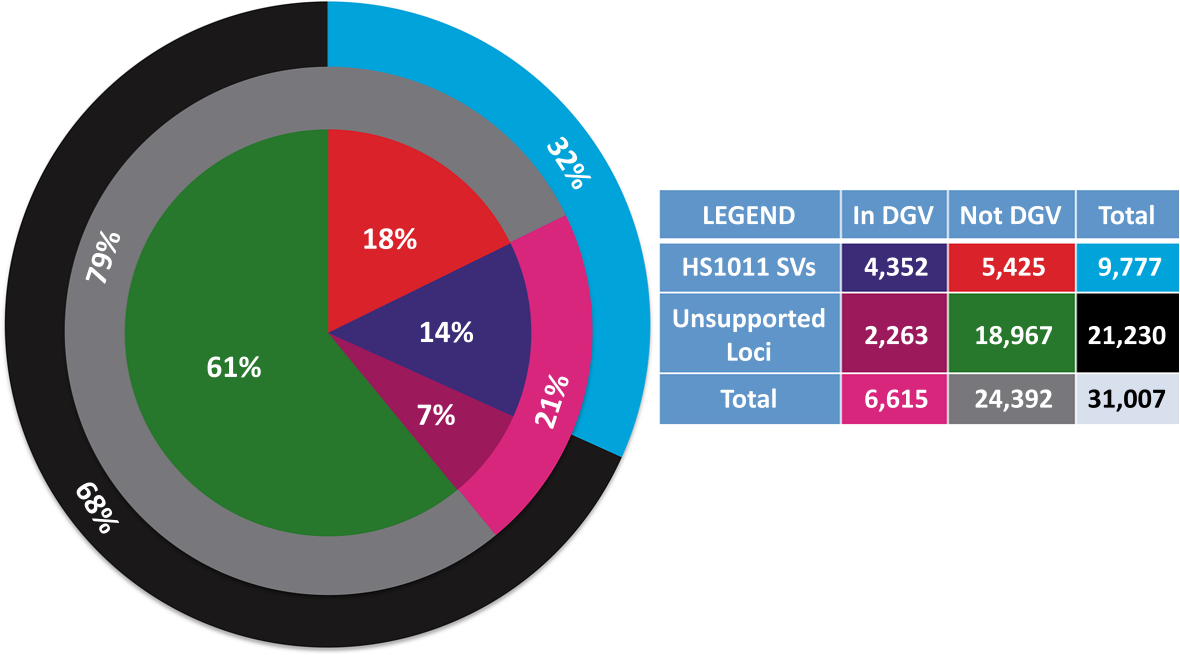
DGV comparison. Each of the 31,007 reference-inconsistent loci was characterized as either an HS1011 SV or unsupported locus based on its Parliament bitflag and as either “In DGV” or “Not DGV” based on whether it shared at least 50% reciprocal overlap with a DGV event of the same type.

Parliament’s integration of multiple data sources and local hybrid assembly provides a systematic assessment of the SV calls made by each program. Each of the 31,007 reference-inconsistent loci was assessed based on three support characteristics, each with a corresponding bit value: assembly support (+4), multi-source support (+2), and long-read force calling (+1). These bit values represent an ordinal prioritization of support types. Combining these bit values for each locus results in a bitflag between 0–7, which provides a compact, extensible, and easily parsed representation of all possible support type combinations. For example, a locus with no support would have a 0 bitflag, while a locus with both assembly and multi-source support would have a bitflag of 6 (4 + 2). The 9,777 SVs comprised all loci with non-zero bitflags. To further understand these SVs, we compared them to known DGV events, runs of homozygous SNV calls, and aCGH data in a family trio. Figure 3 illustrates that of the 9,777 HS1011 SVs, 4,352 matched to a DGV event. The remaining 5,425 HS1011 SVs that did not match a DGV entry reflect either previously uncharacterized SVs or the low resolution of many DGV events. Thus, as with SNVs [31], many SVs in a personal genome represent rare or private variants not observed in databases [11]. We also identified 2,263 unsupported loci that match with DGV events. While these events may correspond to potential HS1011 false negative SVs, they may also be the result of common mapping artifacts represented as variants in DGV or incidental overlap of the DGV events, which cover 70% of the human genome.

**Figure 4:**
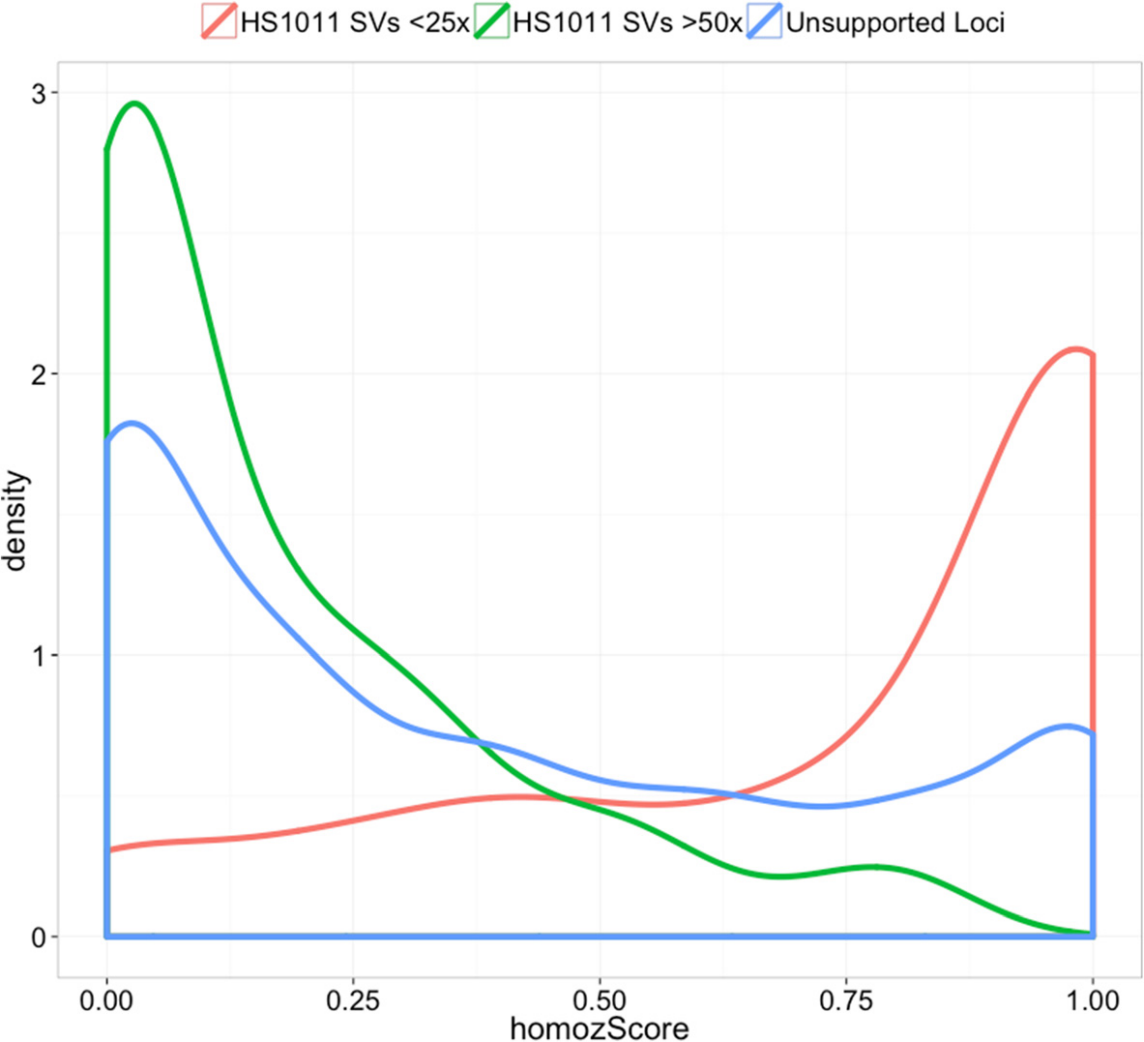
SNP concordance. HomozScores are reported for three classes of HS1011 deletion loci: unsupported loci, HS1011 SVs with less than 25X coverage, HS1011 SVs with greater than 50X coverage.

We next compared the deletion calls to SNVs identified based on WGS Illumina sequencing data by using runs of homozygous SNVs as proxy (homozScore) for deleted regions. HomozScore refers to the fraction of homozygous SNVs in a deleted region (see Methods). We focused on deletions within autosomes that encompassed five or more SNVs. Figure 4 indicates that the supported SV set (i.e., 5,044 deletions with non-zero bitflags) is enriched for deletions consistent with SNV data. For deletions in this supported set that have average coverage less than 25X, we identified 59% (96/161) with homozScore >0.8. In contrast, for the unsupported (i.e., 0 bitflag) deletions only 18% (260/1,426) have a homozScore >0.8, reflecting identification of false positive deletion calls. To provide context for the homozScore, we identified those HS1011 deletions that share 90% reciprocal overlap with a high-confidence set of population deletions from the 1000 Genomes Project [32]. Of these 54 deletions, 52 have a homozScore > 0.8.

To further assess deletion detection and assembly accuracy, we experimentally validated 42 deletions that were amenable to long-range PCR and sequencing. These deletions had an average size of 10.6 kbp (min_size = 3,139 bp and max_size = 53,924 bp; median = 7,613 bp), calculated from exact breakpoints determined by Sanger sequencing. The average difference between the Sanger and Parliament breakpoints for these events was 44 bp, and the median difference was 2 bp (Additional file 1: Table S2). These values may be subject to alignment ambiguities caused by repeat-mediated breakpoints or microhomology generated by rearrangement mechanisms such as Fork Stalling Template Switching (FoSTeS) or microhomology mediated break induced replication (MMBIR) [33,34]. For example, our largest deviation was 1,065 bp for an event mediated by two L1PA5-L1 repeats with 90% identical sequence. We used the same 42 PCR assays to perform family-based analyses using aCGH data from the subject’s parents [25]. Of these 42, all were confirmed in at least one parent and 17 are found in both (Additional file 2: Figures S1 and S2), indicating no *de novo* mutations in this small subset.

### Exonic SV content

We investigated whether SVs were enriched in exonic regions, limiting this analysis to events smaller than 100 kbp to avoid large-event bias. The 31,007 reference-inconsistent loci include 30,573 loci less than 100 kbp that span 88,832,602 bp of the genome (2.7%). Of these loci, 1,859 cover 1,021,515 bp of 5,035 exons (2.8% of the total exonic sequence). Similarly, 9,566 of the supported SVs are less than 100 kbp, spanning 22,898,826 bp (0.7% of the genome). Of these SVs, 293 span 270,537 bp of 1,372 exons (0.7% of the total exonic sequence). These similar values do not suggest exonic enrichment for either the reference-inconsistent loci or the HS1011 SVs. Table 3 lists all genes with exons intersecting an assembly-supported SV (i.e., bitflag >=4) that is not found in DGV. A full list of exons overlapping SVs is provided in Additional file 1: Table S3.

**Table 3:** **Exonic SVs with assembly support absent from DGV**

**Gene**	**Chr**	**Start**	**End**	**Type**	**Source**	**Flag**
ASTN1	1	177,131,105	177,139,508	INS	ARR,ILL,PAC	7
C16orf96	16	4,619,222	4,629,905	INS	ARR,ILL,PAC	7
DOCK3	3	50,879,021	50,879,201	INS	ILL,PAC	7
PCNXL4	14	60,575,443	60,575,546	INS	ILL,PAC	7
PKD1L3	16	72,030,321	72,032,511	INS	ILL,PAC	7
TBC1D3G	17	34,805,579	34,815,084	DEL	ILL,PAC	7
MAGEA11	X	148,735,694	148,830,894	MIS	ILL,NEX	6
METTL21C	13	103,345,467	103,348,148	INS	ILL,NEX	6
RAP1GDS1	4	99,179,171	99,184,420	DEL	ARR	5
ZNF826P	19	20,504,301	20,595,300	DEL	ILL	5
ASTN1	1	177,131,101	177,139,495	MIS	ILL	5
C20orf96	20	258,785	260,245	DEL	ARR	5
CSMD3	8	113,234,275	113,239,237	DEL	NEX	5
GMCL1	2	70,066,401	70,071,781	MIS	NEX	5
HLA-DRB1	6	32,547,848	32,548,158	DEL	ILL	5
MLF1IP	4	185,651,743	185,652,393	INS	ILL	5
MTO1	6	74,203,747	74,216,637	MIS	ILL	5
MUC2	11	1,092,829	1,093,579	INS	ILL	5
OR4C6	11	55,431,550	55,457,289	INS	ILL	5
PDE4DIP	1	144,954,098	144,960,871	MIS	ILL	5
RAB11FIP3	16	544,841	547,158	INS	NEX	5
AP2A2	11	915,248	928,463	INS	ARR	4
TPSB2	16	1,274,089	1,288,819	INS	ARR	4
TPSG1	16	1,274,089	1,288,819	INS	ARR	4
ALDH16A1	19	49,966,580	49,968,737	INS	NEX	4
ASMTL	X	1,550,501	1,572,400	INS	ILL	4
C14orf39	14	60,913,981	60,941,067	MIS	ILL	4
CCL24	7	75,438,336	75,451,936	INS	ILL	4
CD99	X	2,651,401	2,699,500	INS	ILL	4
CNTNAP3B	9	43,844,101	43,866,100	INS	ILL	4
CTNNA2	2	80,769,022	80,780,356	MIS	ILL	4
DEFA1	8	6,833,701	6,844,000	DEL	ILL	4
DSPP	4	88,536,463	88,536,667	INS	ILL	4
ENPP7	17	77,699,565	77,726,581	MIS	ILL	4
EXOC6B	2	72,688,192	72,697,903	MIS	ILL	4
FAM186A	12	50,745,742	50,745,861	INS	PAC	4
FOXO6	1	41,847,826	41,847,932	INS	PAC	4
FRK	6	116,274,618	116,307,096	MIS	ILL	4
HERC2	15	28,547,301	28,566,700	INS	ILL	4
HSD17B3	9	99,057,917	99,063,709	MIS	ILL	4
IFNAR1	21	34,694,683	34,701,442	MIS	NEX	4
IGHV4-61	14	107,087,259	107,099,190	INS	ILL	4
IL28A	19	39,730,613	39,762,849	MIS	NEX	4
IL28B	19	39,730,613	39,762,849	MIS	NEX	4
IL3RA	X	1,494,601	1,510,800	INS	ILL	4
KIAA1671	22	25,441,077	25,467,572	INS	ILL	4
KRT37	17	39,579,211	39,595,476	MIS	ILL	4
KRT38	17	39,579,211	39,595,476	MIS	ILL	4
KRTAP4-7	17	39,240,740	39,240,840	INS	PAC	4
KRTAP5-4	11	1,642,915	1,643,128	INS	PAC	4
MATR3	5	138,652,720	138,666,146	INS	ARR	4
NBPF15	1	148,571,852	148,591,725	INS	ARR	4
OR2A7	7	143,945,501	143,956,800	INS	ILL	4
OR2G6	1	248,682,789	248,702,341	MIS	NEX	4
OR4C3	11	48,340,701	48,347,600	INS	ILL	4
OR4C6	11	55,431,551	55,445,867	INS	PAC	4
OR8U1	11	56,143,129	56,143,999	INS	PAC	4
PLXNB2	22	50,723,862	50,724,455	DEL	ILL	4
PPP2R3B	X	290,201	300,100	INS	ILL	4
PPP2R3B	X	327,401	344,700	INS	ILL	4
PRIM2	6	57,494,250	57,507,908	INS	ILL	4
RFC1	4	39,350,151	39,353,407	INS	NEX	4
RGPD3	2	107,082,401	107,085,300	DEL	ILL	4
RRBP1	20	17,639,769	17,639,981	INS	PAC	4
SAMD1	19	14,200,852	14,200,953	INS	PAC	4
SHOX	X	598,001	628,300	INS	ILL	4
SLC25A6	X	1,494,601	1,510,800	INS	ILL	4
SMC1B	22	45,745,435	45,746,440	INS	ILL	4
TMEFF2	2	192,818,912	192,840,053	MIS	ILL	4
UTS2D	3	190,999,450	191,019,577	INS	ILL	4
XG	X	2,651,401	2,699,500	INS	ILL	4
ZNF208	19	22,156,807	22,156,912	DEL	PAC	4
ZNF253	19	19,990,342	20,005,317	MIS	ILL	4
ZNF346	5	176,462,777	176,474,191	INS	ILL	4
ZNF519	18	14,105,158	14,105,265	DEL	PAC	4

Genomic location, SV type, Parliament bitflag, and supporting data types are provided for the 75 HS1011 SVs overlapping an exon but not matching a DGV event.

### Multi-source comparison

**Figure 5:**
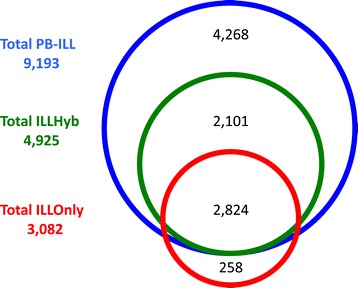
Illumina-Only & PacBio comparison. The Illumina only results are compared to the HS1011 SV subset containing Illumina and PacBio discovery. PB-ILL contains all HS1011 SVs with PacBio or Illumina discovery and hybrid assembly support. The ILLHyb workflow uses only PE methods for discovery but both Illumina and PacBio sequence reads for local assembly. The ILLOnly workflow uses only Illumina PE methods and reads for both discovery and assembly.

To further elucidate the impact of long-read data on SV detection, we developed a parallel Illumina workflow (Figure 1c) that uses only Illumina HiSeq PE data for both SV discovery and local assembly evaluation. This workflow (IllOnly) identified 17,706 reference-inconsistent loci, 3,082 of which were supported by local Illumina-only assembly. We then assessed each of the 17,706 IllOnly loci with local Illumina/PacBio hybrid assembly support. The IllOnly Parliament workflow was 86.41% accurate, 97.92% specific, and 57.34% sensitive, with 2,824 of the 3,082 IllOnly SVs supported by hybrid assembly (Additional file 1: Table S4). We also recovered 2,101 SVs that lacked IllOnly assembly support but were supported by hybrid assembly (Figure 5). Table 4 compares the false-discovery rates (FDRs) and sensitivities of each Illumina HiSeq SV method and the Parliament IllOnly workflow. Parliament is the only method with less than 10% FDR and greater than 50% sensitivity. Despite these benefits of a multi-algorithm approach, Illumina-only discovery still only recovers approximately half of the 9,777 SVs identified by multi-source Parliament: PBHoney alone identifies 4,268 SVs supported by hybrid assembly, representing events “invisible” to PE data.

**Table 4:** **Illumina-only method comparison**

**Program**	**Total called**	**Supported**	**Unsupported**	**FDR**	**Sensitivity**
CNVnator	6,197	1,211	4,986	80.46%	22.62%
BreakDancer	5,520	2,269	3,251	58.89%	42.39%
Delly	3,720	1,669	2,051	55.13%	31.18%
Crest	2,219	1,889	330	14.87%	35.29%
Pindel	4,451	3,035	1,416	31.81%	56.70%
SV-STAT	892	876	16	1.79%	16.36%
Tiresias	1,347	417	930	69.04%	7.79%
Spiral	1,881	1,824	57	3.03%	34.07%
**Parliament**	**3,082**	**2,852**	**258**	**8.37%**	**57.34%**

Performance for each Illumina-only method is summarized. Supported and Unsupported columns indicate the number of calls with and without local hybrid assembly support, respectively. False discovery rate (FDR) and sensitivity are calculated using all 17,704 Illumina Only reference inconsistent loci and the subset of 5,584 that are supported by hybrid assembly.

**Figure 6:**
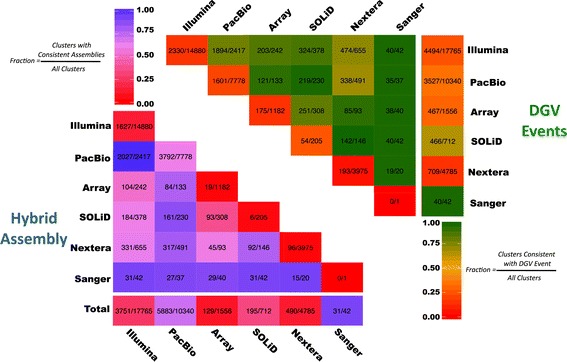
Multi-source comparison. Each cell contains the number of clusters with support from a pair of sources. The diagonal entries describe clusters with support with exactly one data source.

Figure 6 illustrates the performance of all source-pair combinations relative to hybrid assembly and DGV events. Calls supported by only one data source are represented on the diagonals, which indicate that such events were both less likely to match a DGV event or have assembly support. All aCGH events, solo or paired, were more likely than other event types to have a DGV match, despite no clear preference in the assembly data. This disparity is likely a combination of the challenges associated with assembling larger events identified by aCGH and the prevalence of such calls in DGV.

### Parliament on DNAnexus

Implementation of Parliament on local compute requires independent installation of multiple discovery tools and a local assembler, imposing a burden of systems administration and resource consumption. We therefore installed the suite on a cloud-based service via DNAnexus, a commercial middle-ware provider. In this implementation, users need only to upload their data, select the desired tools, and adjust Parliament parameters. Currently, DNAnexus supports a full version of Parliament as well as a lightweight BAM-to-SV workflow that requires only Illumina paired-end WGS data.

All HS1011 data used in this study and the full result set are also available on DNAnexus to facilitate software development and benchmarking. These data and the Parliament infrastructure compose a publicly available resource for developers wishing to evaluate novel SV detection methods in a scalable environment (Figure 1a). Users can either upload their HS1011 result set in Parliament format or create a DNAnexus app to run a newly developed program directly in the Parliament workflow. In either case, Parliament will update the HS1011 reference-inconsistent loci with the novel events and reassemble any new or modified loci with the Illumina and PacBio data, reporting the novel method’s performance relative to the existing data set.

## Discussion

Previous single genome analyses have used combinations of array, unpaired short-read, and PE data to identify large variants [28-30,35-39]. A diploid *de novo* assembly of a single individual (HuRef) identified 8,152,407 bp that are structurally variable when compared to the version 36 human genome reference assembly [37]. However, subsequent application of SV detection methods to array and PE data identified an additional 40,625,059 structurally variant base pairs that, when compared to other genomic characterizations, suggest the limitations of assembly, PE data, and array based SV-detection methods [28].

As the number of personal genomes increases in the clinical setting, overcoming the limitations of SV discovery will be critical for diagnosing genetic disease. Even with the variety of methods and depth of data applied here to HS1011, resolving SVs in a personal genome remains a challenge. While Parliament was designed to provide the most comprehensive set of SVs for a genome given all available data, the HS1011 results also point the way forward to a gold standard SV set. Figure 6 indicates that many assembly-supported calls are made only from short-read or long-read methods, lacking multi-source support. Inspection of several of these events indicates that short- and long-read mappability and long-read coverage could account for some single-source discovery of HS1011 SVs (Additional file 2: Figure S4). To further refine the HS1011 SVs, future Parliament analysis will incorporate additional short- and long-read coverage and short-read libraries of varying insert sizes. These additional data will allow us to better distinguish method-based inaccuracies from the limitations of the data themselves, identify optimal data characteristics for SV discovery, and better characterize existing SVs (e.g., evaluate zygosity, differentiate overlapping but distinct alleles, resolve complex events, eliminate false positives).

Array-based calls present a different set of challenges, as aCGH provides neither orientation nor positional information, but rather reports a value relative to a control and thus can identify CNVs but not copy-number neutral SVs such as inversions. Moreover, a relative gain in a subject could correspond to a loss in the control genome. Finally, gains reported by aCGH do not specify the exact location of those gains, only the portion of the genome that shows the relative gain; clinical aCGH and FISH studies reveal insertional translocations occur 160X more frequently than previously thought [40]. To characterize gains, these duplicated regions would have to be compared to all possible insertion location to confirm the nature of the event. Future work will include incorporating such non-local variant annotation, which will also improve the size estimates of events represented as distant breakpoints.

**Figure 7:**
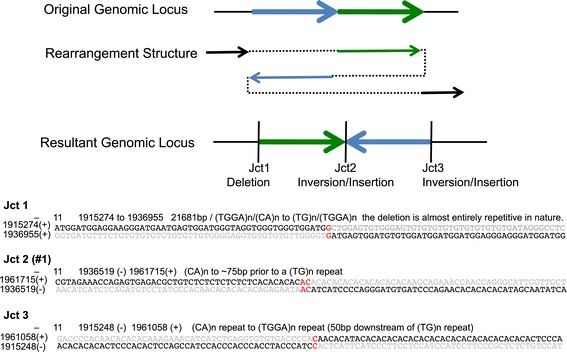
Complex rearrangement. A representation of a large-scale deletion and inverted insertion rearrangement on chromosome 11 p15.5 is depicted. Through *de novo* assembly, the rearrangement breakpoint junctions (Jct 1, 2, and 3) were identified, and the resultant structure in the genome of HS1011 was found to be as depicted. Below are shown the junction sequences of the three breakpoints.

Development of such variant annotation will also include improved resolution of complex genomic rearrangements (CGRs) [41]. Figure 7 describes a CGR in HS1011 identified by PE-only methods as separate events. Manual examination of the macaque (BGI CR_1.0/rheMac3) and chimpanzee (CSAC 2.1.4/panTro4) genomes revealed that the organization of this syntenic locus was similar to that reconstructed from HS1011. In the chimpanzee genome a large gap encompasses the relative position of the deletion event. Examination of a fosmid mapping resource available for hg18 [42] shows that 9 of 9 genomes contain deletion, inversion, and insertion fosmids at this locus. Therefore, the rearrangement found in HS1011 may be the common allele, or the hg19 reference may represent an erroneous sequence at this locus.

While further refinement of the HS1011 data will increase the specificity of the SV set, it is unlikely that high-throughput personal genome SV characterizations will have access to all the HS1011 data types. As with SNVs, we can enrich for SVs of interest by incorporating corroborating data, such as family data, runs of homozygosity, and phenotype-specific gene lists. With this in mind, our Illumina-only SV detection workflow built within the Parliament prioritizes putative SV events based on the degree of support (Parliament bitflag) rather than assigning a threshold for “truth.” Such prioritization is particularly appropriate for SVs considering that any SV with purported clinical impact will likely undergo manual curation or orthogonal validation. Moreover, as reported here, applying multiple technologies and methods to a personal genome still only reveals the tens of thousands of reference-inconsistent loci that could indicate the presence of structural variation. While the calls made by different Illumina HiSeq methods largely overlap, there are 1,601 HS1011 SVs identified by only one method, more than 10% of the total events (Table 2). Considering the millions of potential SNVs whole genome data yield and the desire to recover potentially rare and complex events, SV detection methods can afford to err on the side of sensitivity.

The Parliament workflows described here were designed for single-sample analysis of HS1011, but the Parliament infrastructure is amenable to multi-source data. By customizing the rules for reducing and merging the input data, Parliament can subtract calls common to multiple samples to create tumor-normal or trio analysis Parliament workflows. Given large sample sets, the Parliament discovery step can be performed in parallel on each sample, merging the resultant calls into a “master list” of sites that can be assessed via local assembly in each sample, providing a project-level summary of structural variation across the cohort.

## Conclusions

Decreasing sequencing costs, a diversity of resequencing technologies, and increased availability of powerful computational resources provide an immediate opportunity to examine a broader class of genomic variation beyond SNVs in the exome. The present work identifies upper (4.5%) and lower (1.8%) estimates of the extent of structural variation in a personal genome and characterizes the impact of various resequencing methods. Applying multiple Parliament workflows, we demonstrate that while method integration is optimal for SV detection in Illumina paired-end data, the addition of long-read data can more than triple the number of SVs detectable in a personal genome. The 9,777 SVs identified from the HS1011 data sources represent the first long-read characterization of structural variation in a diploid human personal genome. Moreover, The HS1011 genome is particularly applicable to the challenges facing SV characterization, not for its specific SV content but because of the diversity of data and the ability to collect even more data as new technologies arise. The HS1011 data and the Parliament infrastructure are available via DNAnexus, lowering barriers to evaluate new SV-detection methods. In turn, each method evaluated via Parliament in this data commons further refines the HS1011 SV data set—improving the desired gold standard characterization of SVs in a personal diploid human genome.

## Methods

### Sample data

Tables 1 and 2 provide references for previously collected data and published methods, respectively. Informed consent was obtained for individual HS1011 under protocol H-29697, which is approved by the Institutional Review Board at Baylor College of Medicine. This protocol provides consent to publish the detailed genomic information contained in this manuscript. Sequence data for HS1011 (BioSample SAMN00009513) can be obtained via the SRA database (accession numbers SRX286419, SRX852867, SRX852868, and SRX852869).

### Illumina Nextera

The WGS Illumina Nextera data is 100 × 100 bp mate pair with an average fragment size of 6.5 kbp providing 71X clone coverage and approximately 2X read coverage.

### Pacific Biosciences

Large-insert PacBio library preparation was conducted by following the User Bulletin - Guidelines for Preparing 20 kbp SMRTbell™ Templates (version 2) and Procedure & Checklist - 20 kbp Template Preparation Using BluePippin Size-Selection (version 3) listed in the website (http://www.pacificbiosciences.com/support/pubmap/documentation.html). In brief, a total of 120 ug genomic HS1011 DNA was sheared into 20 kbp targeted size by using Covaris g-TUBEs (Cat.# 520079, Covaris) on Eppendorf 5424 centrifuge. Each shearing processed 10 ug input DNA and a total of 12 shearings were performed. The sheared genomic DNA was examined by Agilent 2100 Bioanalyzer DNA12000 Chip (Cat.# 5067–1508, Agilent Technologies Inc.) for size distribution and underwent DNA damage repair/end repair, blunt-end adaptor ligation followed by exonuclease digestion. The purified digestion products were loaded onto pre-cast 0.75% agarose cassettes (Cat.# BHZ7510, Sage Science) for 7–50 kbp size selection using BluePippin Size Selection System (Cat.# BLU0001, Sage Science), and the recovered size-selected library products were purified using 0.5x pre-washed Agencourt AMPure XP beads (A63880, Beckman Coulter). The final libraries were examined by Agilent 2100 Bioanalyzer DNA12000 Chip for size distribution and the library concentrations were determined by Qubit 2.0 Fluorometer (Cat.# Q32866, Life Technologies).

### BioNano Irys

Cells were washed with PBS, resuspended in cell resuspension buffer, and embedded in gel plugs (BioRad #170-3592). Plugs were incubated with lysis buffer and proteinase K for four hours at 50°C. The plugs were washed and then solubilized with GELase (Epicentre). The purified DNA was subjected to four hours of drop dialysis. It was quantified using Nanodrop 1000 (Thermal Fisher Scientific) and/or Quant-iT dsDNA Assay Kit (Invitrogen/Molecular Probes), and the quality was assessed using pulsed-field gel electrophoresis.

DNA was labeled according to commercial protocols using the IrysPrep Reagent Kit (BioNano Genomics, Inc.). Specifically, 300 ng of purified genomic DNA were nicked with 7U nicking endonuclease Nt.BspQI (New England BioLabs, NEB) at 37°C for two hours in NEB Buffer 3. The nicked DNA was labeled with a fluorescent-dUTP nucleotide analog using Taq polymerase (NEB) for one hour at 72°C. After labeling, the nicks were ligated with Taq ligase (NEB) in the presence of dNTPs. The backbone of fluorescently labeled DNA was stained with YOYO-1 (Invitrogen).

The DNA was loaded onto the nanochannel array of the BioNano Genomics IrysChip by electrophoresis of DNA. Linearized DNA molecules were then imaged automatically followed by repeated cycles of DNA loading using the BioNano Genomics Irys system.

The DNA molecules backbones (YOYO-1 stained) and locations of fluorescent labels along each molecule were detected using the in-house software package, IrysView. The set of label locations of each DNA molecule defines an individual single-molecule map.

Single-molecule maps were assembled de novo into consensus maps using tools developed at BioNano Genomics. Briefly, the assembler is a custom implementation of the overlap-layout-consensus paradigm with a maximum likelihood model. An overlap graph was generated based on pairwise comparison of all molecules as input. Redundant and spurious edges were removed. The assembler outputs the longest path in the graph and consensus maps were derived. Consensus maps are further refined by mapping single molecule maps to the consensus maps and label positions are recalculated. Refined consensus maps are extended by mapping single molecules to the ends of the consensus and calculating label positions beyond the initial maps. After merging of overlapping maps, a final set of consensus maps was output and used for subsequent analysis (Additional file 2: Figure S3).

Alignments between consensus maps were obtained using a dynamic programming approach where the scoring function was the likelihood of a pair of intervals being similar. Likelihood is calculated based on a noise model which takes into account fixed sizing error, sizing error which scales linearly with the interval size, misaligned sites (false positives and false negatives), and optical resolution. An interval or range of intervals whose cumulative likelihood is worse than 0.01 percent is classified as an outlier region. If such regions occur between highly scoring regions, an insertion or deletion call is made in the outlier region, depending on the relative size of the region on the query and reference maps.

At present, the BioNano assembly approach is agnostic to allelic bias when calling SVs, and all Irys SV calls are presumed to the homozygous. The BNG *de novo* assembly approach chooses a single haploid when representing a flattened reference model (which is the current BNG standard), presumably the allele that is present in the majority of molecules.

### Array comparative genomic hybridization

Genomic DNAs of all four members of the family were utilized to perform aCGH using a variety of high-resolution platforms to detect CNVs in the family quartet. NA10851 DNA obtained from a cell line from Coriell Cell Repositories (http://ccr.coriell.org) was used as control for the comparative genomic hybridization for all individuals and platforms.

### Agilent 1 M whole-genome aCGH

Array comparative genomic hybridization (aCGH) using Agilent’s 1 Million whole-genome high-density oligonucleotide microarrays containing one million probes across the genome was performed in the four members of the quartet and two additional siblings. Briefly, samples and control DNAs (2500 ng) were digested with the enzymes *Alu*I and *Rsa*I. Following digestion, the sample DNAs were labeled with Cy5-dCTP and the control DNAs were labeled with Cy3-dCTP using the BioPrime Array CGH genomic labeling kit (Invitrogen Corporation, Carlsbad, CA, USA). Purification of and quatitation of the labeled genomic DNA was performed and samples and controls were matched accordingly. Sample plus control labeled DNAs were mixed with human Cot-I DNA for blocking unspecific hybridization and mixed in blocking and hybridization buffers according to the manufacturer’s protocol. After pre-hybridization incubation, the labeled DNAs were deposited on the 1 M array slide for competitive hybridization to take place for 40 hours at 65°C. Washing, scanning, and data feature extraction were conducted according to the manufacturer’s protocol.

### NimbleGen 4.2 M whole-genome aCGH

NimbleGen’s 4.2 Million whole-genome array platform was also used on the four members of the quartet. Briefly, genomic DNAs for samples and control (0.5 ug) were labeled using the manufacturer’s Cy3 (test sample) or Cy5 (control) labeled random nonamers. Labeled products were precipitated, purified and combined (sample + control) for competitive hybridization on the array slide at 42°C for 72 hours. After hybridization, washing, scanning, image processing and data extraction were conducted according to the manufacturer’s protocol and software.

### PCR amplification and sequencing of breakpoints

Specific PCR primers based on the aCGH and SOLiD sequencing CNV calls were designed. Standard end-point and long-range PCR reactions were performed in order to amplify the specific CNV breakpoints. Sanger sequencing was done on all of the successfully amplified PCR products in order to elucidate the specific sequence and coordinates where the breakpoints occurred.

### Software

Structural Variation detection by STAck and Tail (SV-STAT) is a reference-guided assembler that detects and ranks SVs at nucleotide resolution. First the algorithm catalogs candidate breakpoints, the genomic coordinates and orientations of which are determined by recurrent partial alignments, or “stacks.” Next, SV-STAT generates a fasta-formatted library of candidate junctions by concatenating breakpoint regions in orders and orientations consistent with otherwise discordant read-pairs. The algorithm’s metric for a candidate is a function of the difference between the scores of alignments A and B, where A is the alignment between a “stacked” read and the reference, and B is a re-alignment of the same read to the candidate junction. Full details will be reported elsewhere. For the purposes of this study, SV-STAT used the predictions of BreakDancer to determine the paired genomic regions in which to search. In this way, SV-STAT provided a ranking of BreakDancer predictions according to the support available at nucleotide resolution. The source code for SV-STAT is publically available (https://gitorious.org/svstat).

Structural Variation Assessment of CHRomosomal Aberrations (SVachra) is a breakpoint-calling program that uses discordant mate pair reads consisting of both inward and outward facing read types, for example, the data delivered by Illumina mate pair and Nextera Tagmentation sequencing libraries. The SVachra program calculates the distributions of the inward and outward facing mate pair types and applies independent clustering of the inward and outward facing discordant mapped reads to call chromosomal aberrations. Both inward and outward facing reads contribute to the calling of SV, reporting. SVachra calls large insertions-deletions, inversions, inter- and intra-chromosomal translocations, reporting breakpoints in the inward facing orientation thereby eliminating the contradictory outward facing read orientations. SVachra Source code is available at http://github.com/oliverhampton/SVachra.

Tiresias identifies mobile element insertions using clusters of improperly mapped read pairs comprising one read that maps uniquely to the genome and one that maps to a set of element-specific consensus sequences. Breakpoints consistent with each cluster are then identified as local genomic positions with multiple termini of soft-clipped reads.

Data mapping and alignment of the original SOLiD WGS sequence data were performed using Life Technologies’ (former Applied Biosystems) SOLiD Software and Corona Lite suite. Illumina WGS data were mapped and aligned using the BCM HGSC Mercury pipeline (See Appendix 3). All other additional analyses were performed using custom Perl scripts for data parsing, comparison, extraction and intersection.

### Standardization

To compare the SV calls made by each program, all calls are first reduced to one of three types: deletion, insertion, and mismatch. Deletions correspond to any regions in the sample that are missing sequence that is locally present in the reference, insertions are regions with more sequence than the reference, and mismatches contain different sequence than the reference (e.g., inversions). Reduced results are stored in a VCF-like format (http://sourceforge.net/projects/parliamentsv/), and these files are then loaded into a SQLite database and clustered in a method- and data-sensitive manner.

Events with reference sequence spans (deletions and mismatches) are clustered using source-specific minimum reciprocal overlap thresholds. For example, the Illumina-based BreakDancer and CNVnator programs both generate calls of similar precision and such calls are grouped if they possess >50% reciprocal overlap. However, Bionano Irys calls contain only outer boundaries, while Crest calls contain exact breakpoints, so the Irys-Crest threshold is 20% reciprocal overlap. Exact breakpoint resolution of zero reference-span events can be complicated by genomic repeats and microhomology of the inserted sequence, resulting in non-overlapping insertion calls of the same event. To account for this ambiguity, insertion events are mean-shift clustered at several scales, with calls from more precise programs requiring clustering at smaller scales. All clustering and merging parameters can be adjusted by the user. A full list of the default parameters can be found in Supplemental Methods.

The BioNano-Irys platform provides outer-boundaries of reference spans with cumulative sequence-length differences between the sample compared to the reference. This differs from other SV detection methods in that a single Irys call may represent multiple individual events. For example, a 10 kbp span in the reference with a sequence-length difference of 1 kbp could represent an insertion event of 2 kbp and a deletion event of 3 kbp. Irys insertion calls have a mean and median span to sequence-length difference of 12.4 kbp and 7.7 kbp. Similarly, deletion calls have a mean and median difference of 18.8 kbp and 13.3 kbp. In order to appropriately incorporate these broad outer-boundaries and nuanced SV definition, a standard reciprocal overlap or mean-shift threshold is not an optimal use of the data. Therefore, we manually inspected the 852 Irys calls against our merged SVs in order to annotate the SVs as having additional support provided by Irys.

Spiral Genetics’ Anchored Assembly performs whole read overlap assembly on corrected, unmapped reads to detect SNVs, indels, and structural variants. Sequencing errors are corrected by generating k-mers from reads and giving each unique k-mer a quality score. Low-scoring k-mers are discarded as erroneous. The set of high scoring, or true k-mers is used to construct a de Bruijn graph representing an error-free reconstruction of the true read sequences. Each read is corrected by finding the globally optimum base substitution(s) so that it aligns to the graph with no mismatches and differs by the smallest base quality score from the original read. Of these corrected reads, those that do not match the reference exactly are assembled into a discontiguous read overlap graph to capture sequence variation from the reference. Variants are mapped to human reference coordinates (GHCr37.p7) by walking the read overlap graph in both directions until an “anchor” read, where a continuous 65 bp matches the reference, denotes the beginning and end of each variant. Where a variant has more than one anchor, pairing information is used to determine the correct location of the anchor. This analysis includes only variants where a variant was classified as a deletion, insertion, tandem repeat, or inversion and anchors on both ends mapped uniquely to the reference. Other variants detected using Anchored Assembly were not included.

### Annotation

Parliament annotates each putative variant with Ensembl gene boundaries, UCSC gene features (e.g., exon, intron, UTR), hg19 gap features (telomeres and centromeres), known variants from DGV, and known repeats from the UCSC repeat masker track. Known variants are matched to putative sites if they have at least 50% reciprocal overlap.

### Hybrid assembly and force calling

The Illumina WGS and PacBio data within 2,000 bp of each variant locus is extracted and locally assembled with PHRAP. After mapping the resulting contigs back to the reference with Blasr, we determine whether the remapped sequence is consistent with the size and type of the corresponding predicted SV event. We classify such matches as “valid” SV events. However, local assembly does not always yield contigs and nor does it always produce variant alleles. Thus, we also “force call” events at every variant locus using the PacBio data, requiring only one PacBio read to be consistent with the predicted SV event.

### SNP concordance

If a deletion occurs within a region that is unique in the genome, we expect all the SNPs in the deleted region to be homozygous. For each deletion locus, we calculate the fraction of homozygous SNVs in a predicted deletion region (homozScore). We include only deletions that have at least 5 SNVs in the region (1,633/17,665). We use the average coverage in the region of <25X (1/2 of the average HS1011 Illumina coverage) to focus on deletion regions likely to be unique in the HS1011 genome: average coverage >50X might indicate a paralogous region in HS1011 genome. Since reads from paralogs sometimes map to the same reference region, they would result in heterozygous SNVs even if the deletion is present. We use homozScore of 0.8 instead of 1 to account for SNV genotyping errors and potential imprecision of SV breakpoints.

## Additional files

Additional file 1:
**Tables S1–S4, describing the full HS1011 SV data set, breakpoint comparison, SV/exon intersection, and performance summary for the Illumina-Only Parliament workflow, respectively.**
Additional file 2:
**Figures S1–S3, describing PCR amplification of CNV breakpoints and segregation in a family quartet,** ***de novo*** **CNV analysis, and single molecule assembly, respectively.**
