# Supplementary material for: Assessing structural variation in a personal genome—towards a human reference diploid genome
Source: BMC Genomics. 2015 Apr 11;16(1):286. doi: 10.1186/s12864-015-1479-3 (PMC4490614; doi:10.1186/s12864-015-1479-3)
Supplement: Additional file 2: — Figures S1–S3, describing PCR amplification of CNV breakpoints and segregation in a family quartet, de novo CNV analysis, and single molecule assembly, respectively. [file 12864_2015_1479_MOESM2_ESM.pdf]

Supplemental Material

Supplemental Table 1 Key:

Chrom, start, end: hg19 coordinates for each event

Svtype: INS(ertion), DEL(etion), MIS(match)

Size: Event size determined from constituent calls' predicted size. May differ from end-start.

Bitflag: integer ranging from 0 to 7 indicating type(s) of support. Events with bitflag > 0 are members of the SV set.

Datatypes: types of data supporting the call

Rsids: indicate supporting methods

RSID	Program/Source
0	BreakDancer_1.4.3
1	CNVnator_v0.2.7
2	Crest_1.0
3	Delly_v0.3.3
4	Pindel_v0.2.4t
5	SV-STAT-0.0.5-vb10
6	Tiresias
7	SprialGenetics
8	Honey
9	SVachra-v1.7
10	aCGH (all)

Varanno: DGV annotation

Geneanno: Encode gene annotation

Genefeatanno: Encode gene feature annotation

Gapanno: gap annotation

Irys: support by bionano Irys data

Supplemental Figure 1 PCR amplification of CNV breakpoints and segregation in a family quartet.

Supplemental Figure 2 Identification of (not) 'de novo' CNVs.

Supplemental Figure 3 BioNano Irys Single Molecule Assembly

Supplemental Figure 4 Example Read Pileups

Supplemental Figure 1 PCR amplification of CNV breakpoints and segregation in a family quartet.

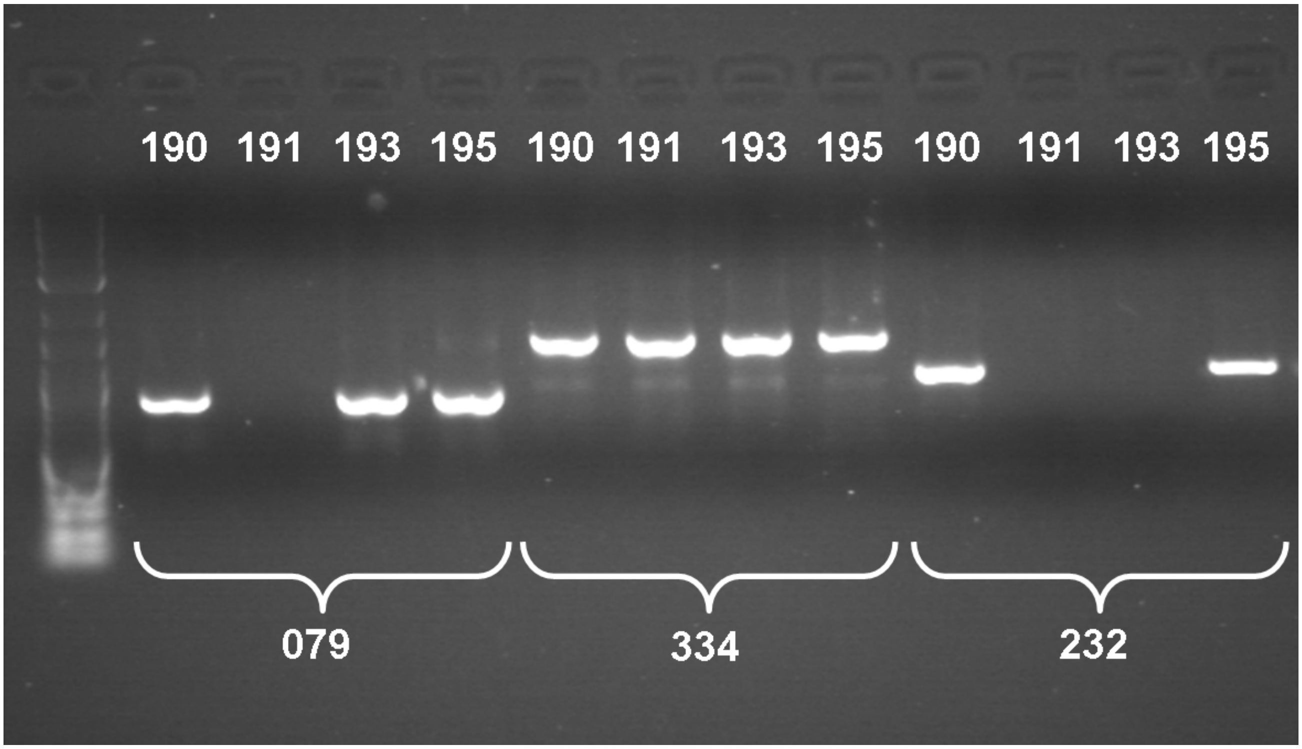

Amplification and segregation of CNV breakpoints by PCR in a family quartet. The first four lanes show segregation and transmission of the CNV079 from parent BAB190 (mother) to the two siblings (BAB193 and BAB195). CNV334 shows that all individuals in the family are carriers of this particular deletion. CNV232 is a 53,924 bp deletion inherited from the mother to the proband BAB195; this deletion encompasses two exons of the *XIRP2* gene.

Supplemental Figure 2 Identification of (not) 'de novo' CNVs.

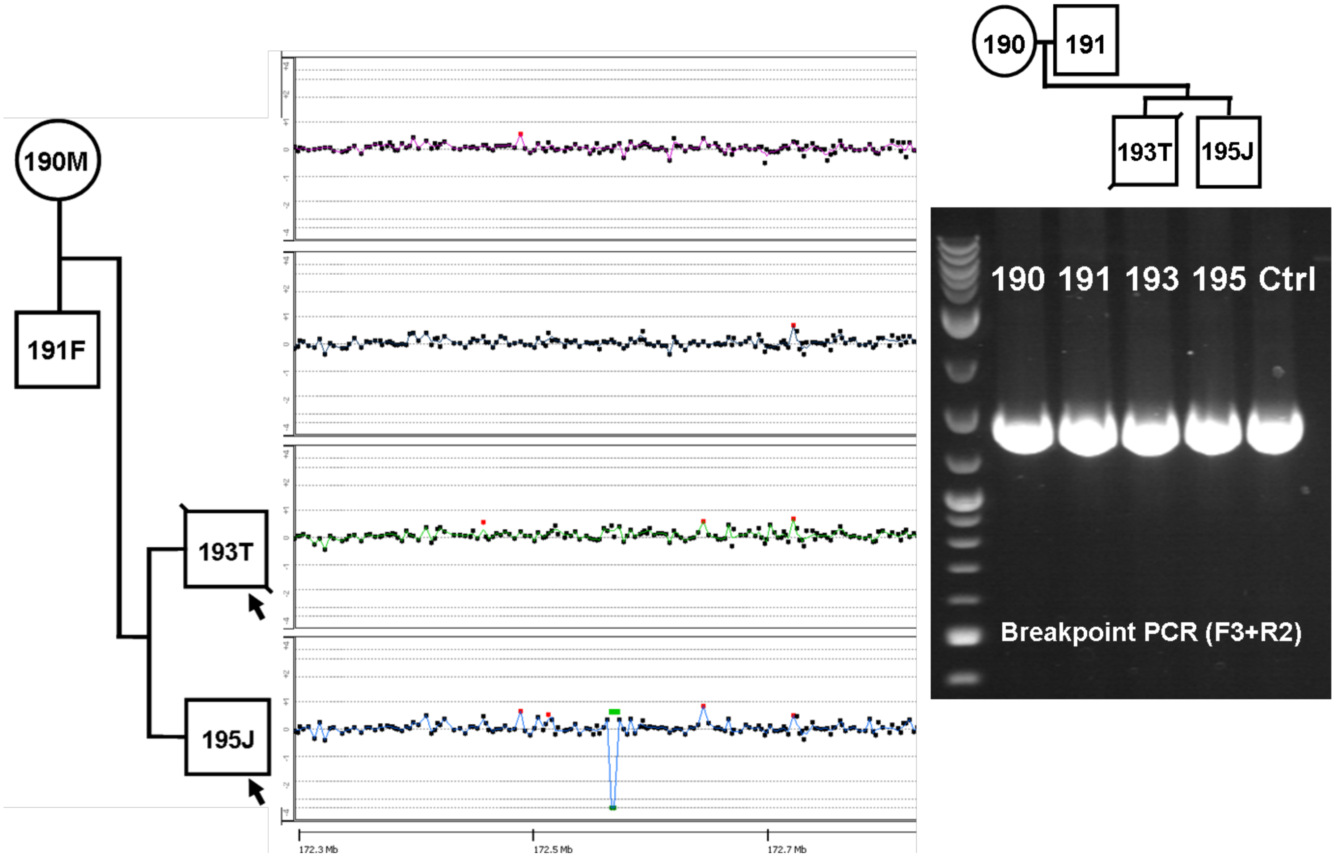

Some CNVs identified by aCGH appeared to be de novo events in one of the offspring, however PCR amplification and segregation of the breakpoint in all the family members and the same control used in the aCGH experiments reveals that this is in reality a polymorphic CNV present in all the individuals and an artifact of the comparative genomic hybridization between the test and control samples.

Supplemental Figure 3 BioNano Irys Single Molecule Assembly

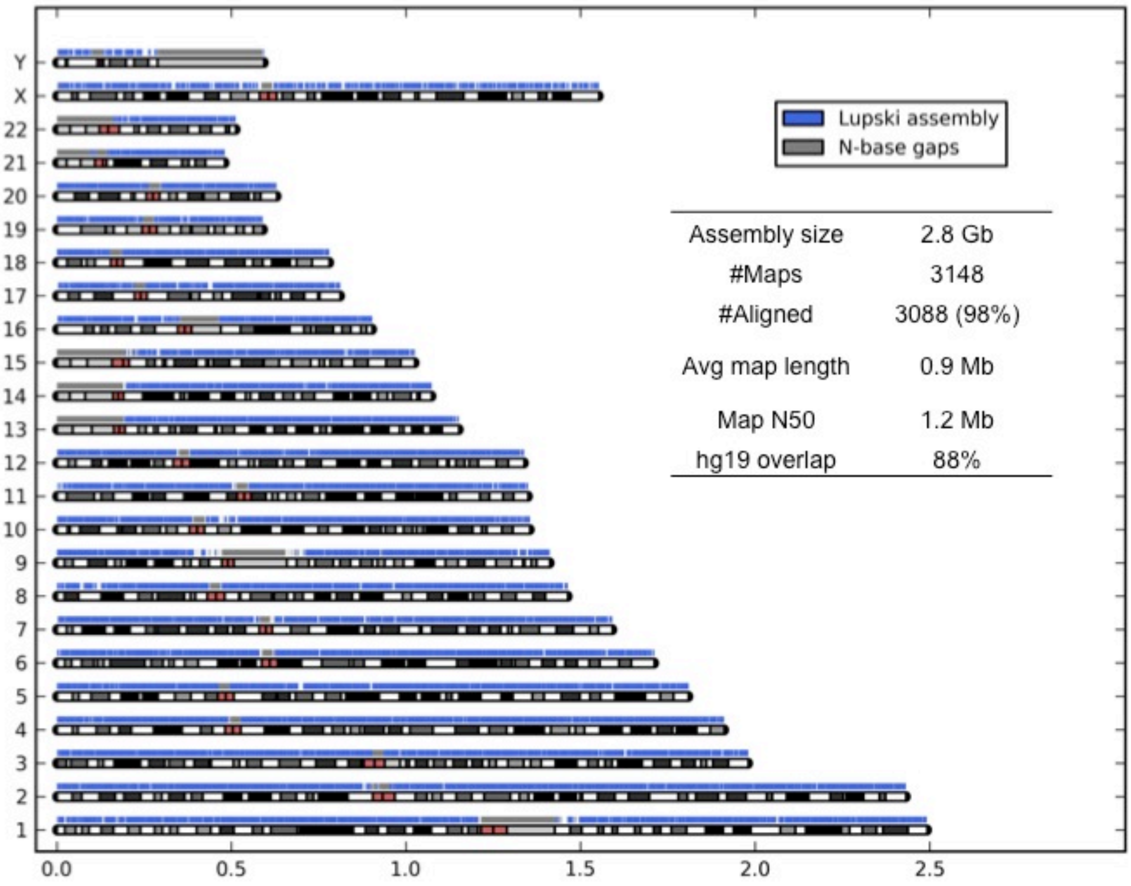

A total of 193 Gb of single molecule data was collected on 4 Irys chips over a 4 day period; 585,908 single molecules were images with an average of 9.57 Nt.BspQI labels per 100Kb. The total set of single molecules were then subject to de novo assembly, of which 483,790 (83%) single molecules participated in the final de novo assembly, producing a total of 3148 genome maps with an average genome map length of 0.9 Mb, N50 of 1.2 Mb, a total assembly size of 2.8 Gb, and hg19 coverage rate of 88%. The average single molecule coverage depth in the de novo assembly is 51.86 X.

Supplemental Figure 4a Read Pileups

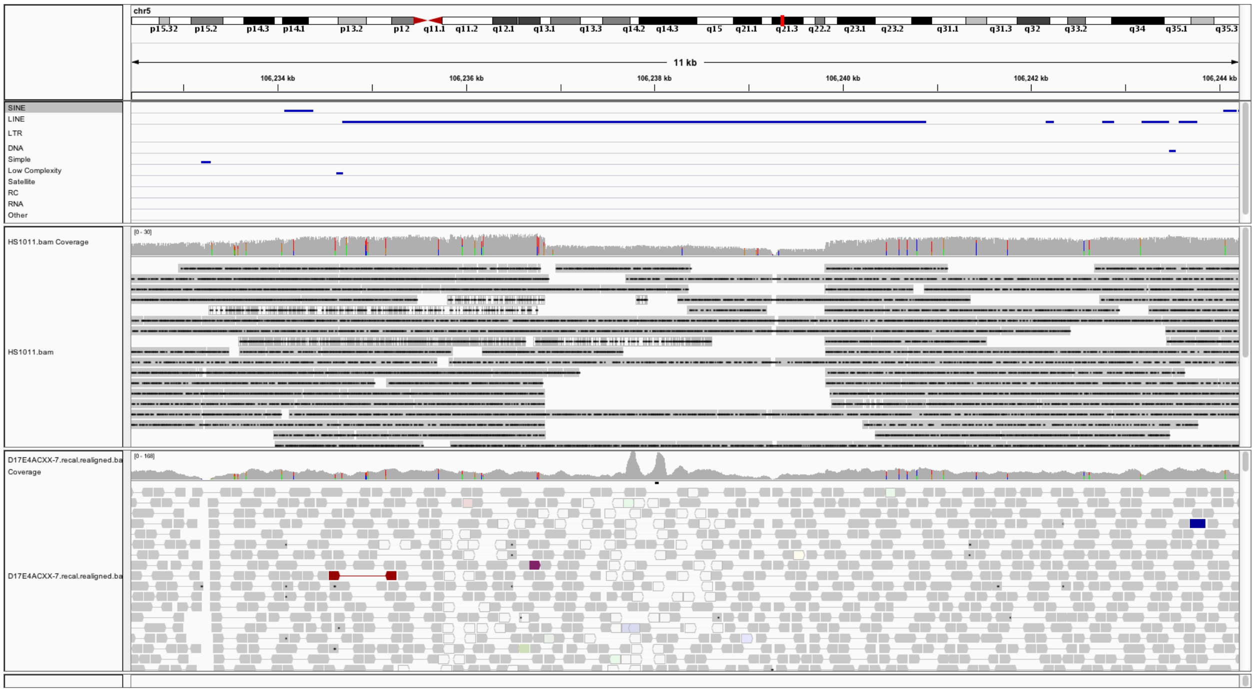

This even is DGV-known heterozygous 2kb deletion that was only discovered using PBHoney (no Illumina methods identified it), but it was assembled through both the hybrid assembly and Illumina-only assembly pipelines. The main portion of the deletion is a LINE element type L1PA3. This region has a low average mappability.

Supplemental Figure 4b Read Pileups

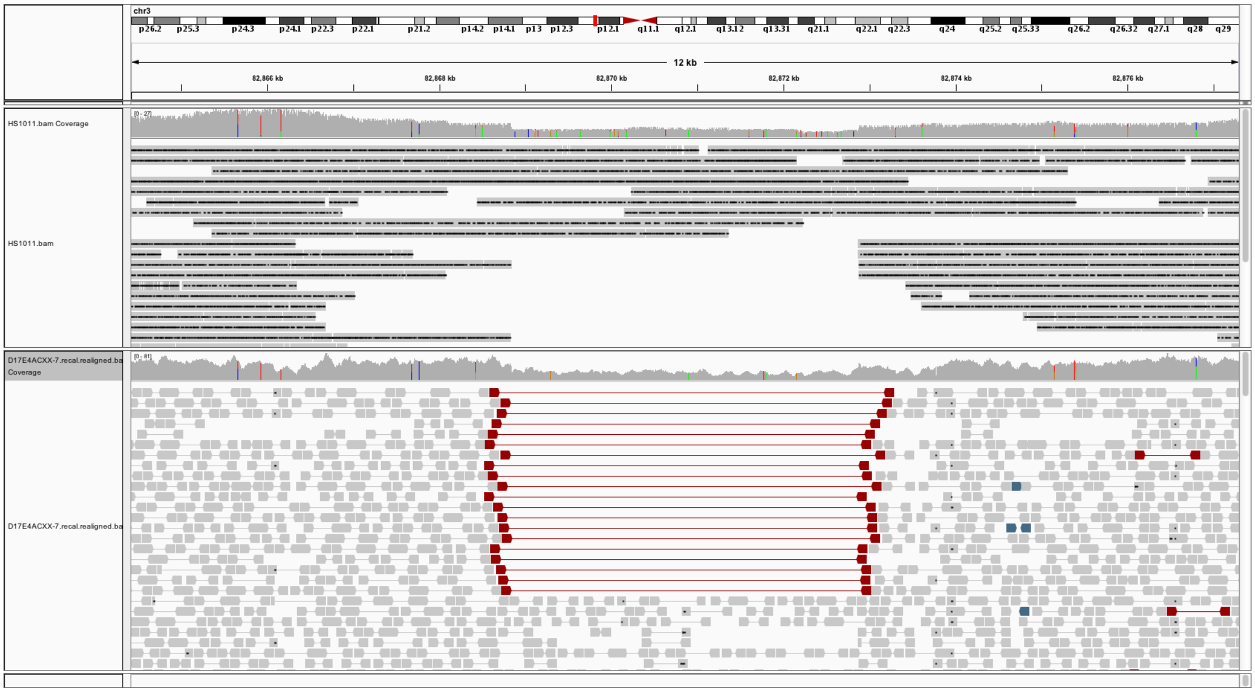

Only Tiresias (the mobile element caller) and PBHoney did not call this ~4 Kbp heterozygous deletion. PBHoney requires a minimum of 3 reads to report an event during the discovery phase. PBHoney force-calling of PacBio reads found evidence supporting the deletion. This event is an example of how lower coverage PacBio reads can work in conjunction with Illumina reads via hybrid-assembly and force-calling even when the PacBio coverage is insufficient to make a call during the discovery phase.

Supplemental Figure 4c Read Pileups

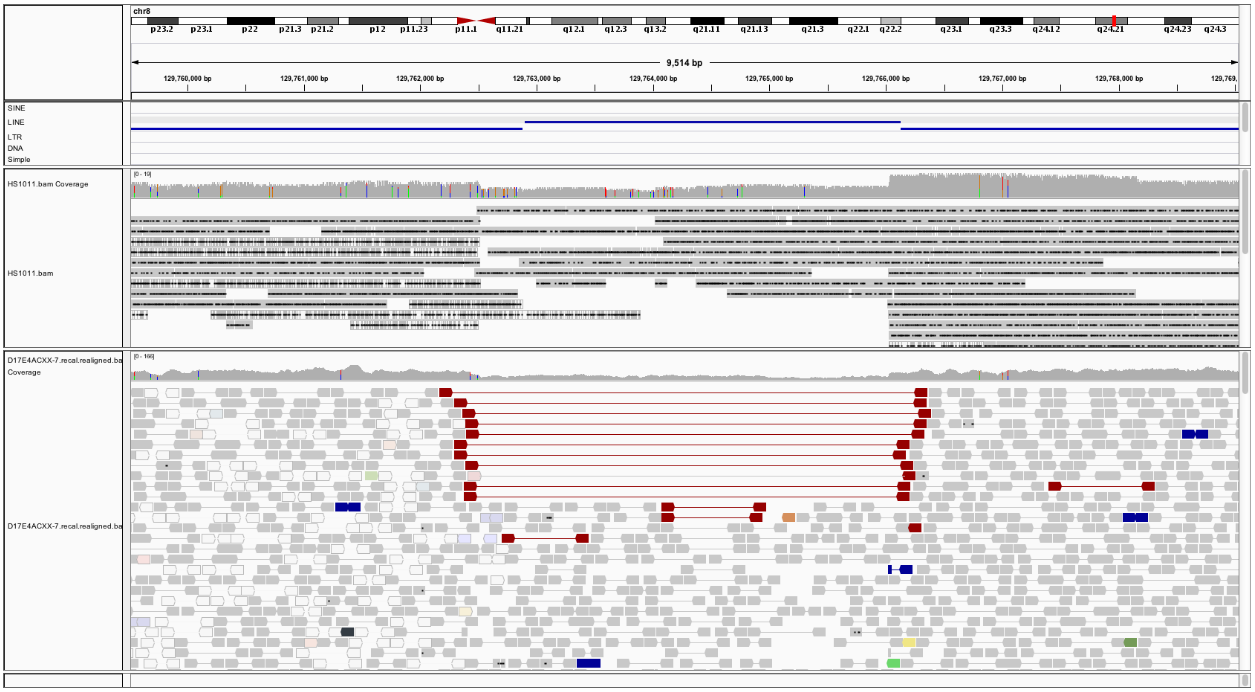

Only the Illumina programs CNVnator, Crest, and Pindel discovered this ~3.5kb heterozygous deletion, which is in a neighborhood of LINE elements. When we remove the minimum MapQ threshold for PBHoney tails from 150 to 0, we find that there are 5 reads supporting the event, one of which passes the mapping quality filter. This event is an example of how individual PacBio reads are not necessarily immune to mapping ambiguities.
